# Identifying key morphometrics to post-storm beach recovery through explainable AI

**DOI:** 10.1038/s41598-024-64023-6

**Published:** 2024-06-20

**Authors:** Salika Thilakarathne, Takayuki Suzuki, Martin Mäll, Masayuki Banno

**Affiliations:** 1 Department of Civil Engineering, Yokohama National University, Yokohama, 240-8501 Japan; 2 Coastal and Estuarine Sediment Dynamics Group, Port and Airport Research Institute, Yokosuka, 239-0826 Japan

**Keywords:** Ocean sciences, Physical oceanography, Natural hazards

## Abstract

In the context of ongoing discussions about climate change, the focus on beach resilience has gained significant attention in contemporary studies. However, a comprehensive understanding of beach resilience, particularly in the short term, remains limited. This study utilizes a dataset of 104 storm events in Hasaki beach, located on the East coast of Japan, to investigate the 10-day beach recovery. The study considers four types of distinct beach profile patterns based on sandbar formations. Employing XGBoost and the SHAP explanation method, the influence of morphometric indicators on beach resilience were explored. Resilient beach profiles are anticipated to exhibit rapid recovery following erosional storm events. The results reveal that morphometrics play a crucial role in determining the short-term, 10-day, recovery of beaches, with specific morphometric features demonstrating pronounced effects based on profile patterns. The study contributes to the current knowledge of post-storm beach recovery and provides insights that could inform discussions on beach resilience.

## Introduction

The beach stands as one of the most dynamic morphological features in the coastal zone^1^. Composed of unconsolidated materials, beaches undergo recoverable changes in response to extreme weather conditions^2,3^. These recovery processes operate across storm-scale to decadal time scales, revealing a cyclic behavior in the profile changes of most beaches^4,5^. Sediment sources from rivers, cliffs, and offshore areas play a crucial role in nearshore sediment balance, acting as key factors in the long-term recovery process. Human activities, including mining and disruptions to sediment supply in fluvial networks, play a crucial role in shaping beach evolvements. Consequently, human interventions, such as artificial nourishment, become essential to sustain the beach system when natural recovery rates are insufficient, ultimately safeguarding its coastal system. Although numerous studies have delved into the cyclic behavior of beach systems across different time scales, the primary focus has been on seasonal and annual time scales. Yet, the influence of coastal storm events brings about substantial changes in surf zone morphology.

Coastal storms are associated with intensified wave actions, leading to significant disruptions within their coastal systems. Castelle et al.^6^ have discussed how storms with varying energy levels result in different erosional patterns in the subaerial beach and contribute to sandbar migration. Moreover, during storms, the infragravity band and mean offshore transport components can be identified as the primary contributors to beach erosion^7^. Winter storms, in particular, have inflicted nearly irreparable damage on many beach systems, leaving coastal engineers grappling with the estimation of recovery times and rates. However, beach erosion caused by single storms differs from that caused by consecutive storms, as erosion volumes per storm depend on the total energy of that storm and preceding storms, with the antecedent morphological state initially being the dominant factor controlling beach response^8–12^. The consequences of coastal storms are far-reaching, posing notable challenges to the coastal environment and surrounding socio-economics^13,14^. Recognized as a vital external element driving morphological changes within nearshore coastal systems, coastal storms play a crucial role in shaping coastal landscapes^15^.

Different coastal sites often exhibit distinct beach recovery patterns, emphasizing the importance of site-specific study approaches^16^. Maspataud et al.^16^ further suggest that temporal and spatial variations in bar-trough beach morphology may also contribute to short-term beach recovery behavior. Focusing on the post-storm beach recovery, Morton et al.^3^ categorized the process into four stages: berm reconstruction and fore-beach accretion, back-beach aggradation, dune formation, and dune expansion, and vegetation recolonization. However, their conceptualization predominantly addresses longer time scales. In the present study, we specifically focus on the storm-scale recovery of the fore-beach accretion; the initial stage of their post-storm recovery model. Despite the disruptive impact of stronger waves on nearshore morphology, the beach’s recovery from such disturbances is facilitated by milder short-period waves in the post-storm period. Surf zone alterations during high wave conditions are primarily attributed to disturbances to the seabed. Instances of high turbulences in the surf zone may induce a seaward net sediment flow, often resulting in the displacement of foreshore sediment further seaward. When examining the beach recovery, a semi-empirical criterion proposed by Sunamura and Horikawa^17^ serves to quantify beach response in this context:

1 $$\begin{aligned} \frac{H_0}{L_0} - C_s \tan (\beta )^{-0.27} \left( \frac{d}{L_0}\right) ^{0.67}\ge 0; erosion \end{aligned}$$

where $H_0$ and $L_0$ are deep-water wave height and length, respectively, $\tan \beta$ is bottom slope, $d_{50}$ is grain size, and $C_s$ is a dimensionless constant. $C_s$ is classified as follows.

$$\begin{aligned}&4< C_s < 8 \quad{} & {} \text {-- erosion and accretion of laboratory beaches} \\&C_s \approx 18\quad{} & {} \text {-- for natural beaches} \end{aligned}$$

Positive values resulting from this calculation indicate erosion. While negative values show beach accretionary conditions.

A dimensionless parameter, $K_*$, proves useful in explaining the stage movement of cross-sectional beach profiles by incorporating wave data and grain sizes^18,19^:

2 $$\begin{aligned} K_* = \frac{\overline{H}_B^2}{g\overline{(T^2d)}} \end{aligned}$$

where $\overline{H}_B$ is daily average breaking water height, d is the sediment grain size, and *T* is the wave period. Based on the $K_*$ value, profile characteristics range from erosional extremes to accretionary extremes. However, these expressions have been derived empirically, and their validity is limited to the conditioned used to derive them. Therefore, there remains a need for sound theoretical equations to address knowledge gaps and provide a clearer understanding of beach recovery mechanisms. While numerous studies have concentrated on hydrodynamic conditions, only a limited number have considered morphological variables related to sandbar characteristics when formulating mathematical equations to comprehend beach behavior in the post-storm period^12,20^.

Existing post-storm recovery quantifications heavily rely on numerical simulations, which often impose a significant computational burden. However, recent advancements in Machine Learning (ML) algorithms offer a promising alternative, showcasing their ability to grasp intricate natural processes with considerably lower computational costs. While empirical equations, predominantly derived from laboratory experiments, aim to understand inter-correlations between major morphometrics and hydrodynamic conditions, improved ML models with extensive hyperparameters have showcased potential in capturing these intricate natural behaviors. The integration of ML in coastal and ocean engineering is currently flourishing, yielding results suggesting higher accuracies in capturing complex relations^21–27^. At the same time, recent developments in data observation techniques, such as remote sensing, have generated large-scale datasets across various scientific disciplines. These datasets are instrumental in training ML or Deep Learning (DL) models to serve as local and global interpretations, explaining dependent variables in respective studies. While DL algorithms trained on neural networks excel in capturing natural behaviors, their performance with numerical tabular data is less compelling^28,29^. In this context, XGBoost^30^, a boosting decision tree algorithm, has proven its excellence in discerning complex relations within homogeneous tabular data. A widely discussed drawback of using machine learning tools is their low explainability; however, recent developments in feature importance tools, such as SHapley Additive exPlanations^31^ (SHAP), have shown effectiveness in interpreting model results^25,32,33^.

Given the limited discussions on the influence of morphometric variables in beach recovery, the present study aims to fill this gap by setting quantitative objectives and employing innovative ML-driven tools for measurement of the role of morphometrics on recovery. Additionally, while the presence of sandbars significantly shapes beach profiles during recovery, relevant studies are notably scarce. To address this, we examine the resilient behavior of four types of distinct profile patterns based on sandbar formations, a consideration often overlooked in prior studies, whether they be erosional or accretionary. By incorporating sandbar formations into our beach resilient discussions, we expect to strengthen the present understanding of the role of beach morphology in its recovery.

## Methods and datasets

### Study site

**Figure 1:**
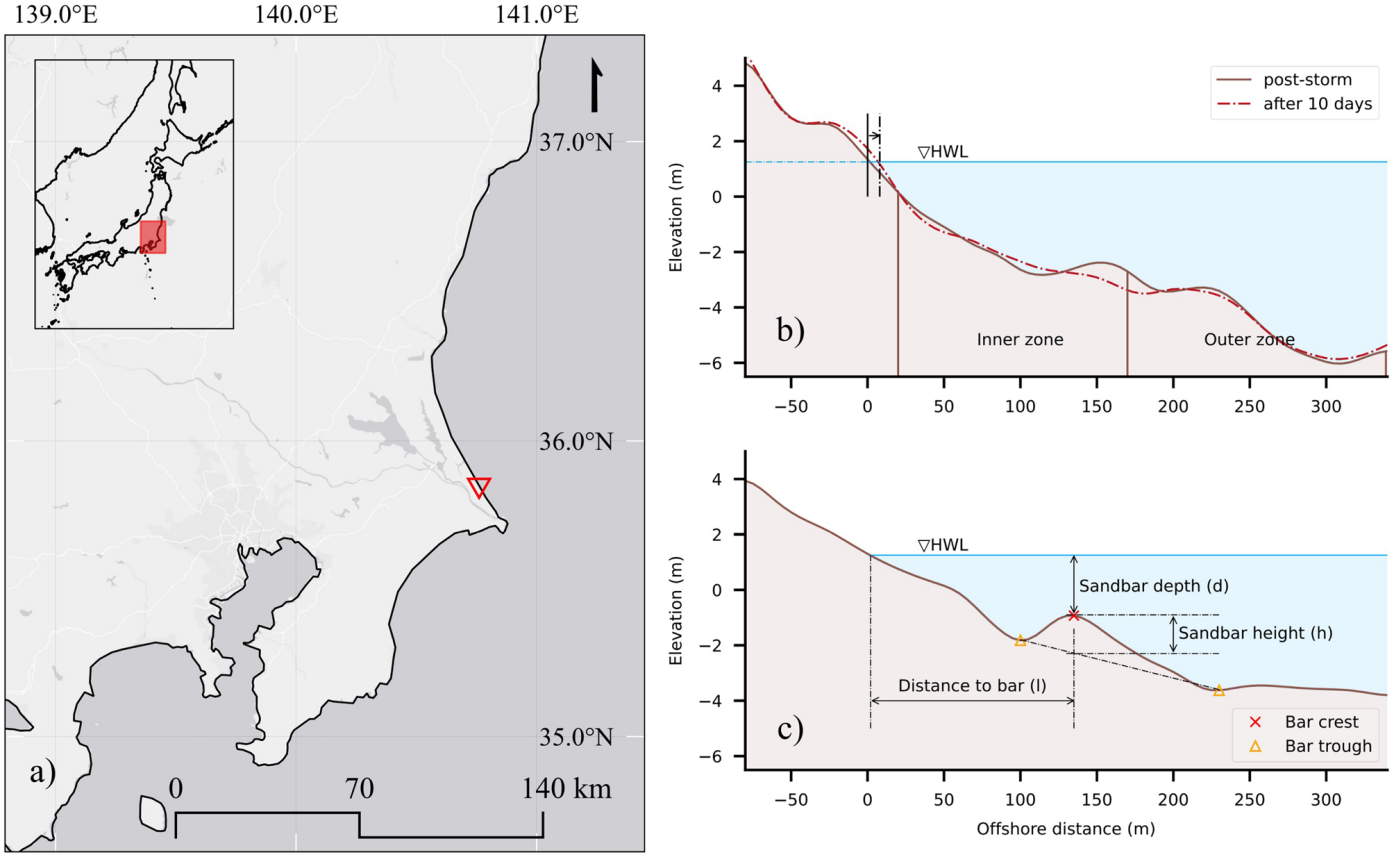
(**a**) Location of the Hasaki Oceanographic Research Station (HORS) in the Ibaraki prefecture, Japan. (**b**) Beach profile immediately after a storm event and after a 10-day period, where shoreline changes based on the high water level (HWL) are considered as the beach recovery. Inner and outer zone definitions are based on the foreshore boundary and average wave breaking location. (**c**) Sandbar identification based on trough and bar formation, with sandbar indicators defined based on this figure.

The Hasaki Oceanographic Research Station (HORS) is situated in the middle of an approximately 16 km long straight sandy beach strip, bounded by Kashima port to the North and Choshi port to the South, in Japan (Fig. 1a). HORS features a 427.0 m long pier extending perpendicularly to the Hasaki coastline. This pier has played a significant role in amassing a valuable dataset documenting morphological and hydrodynamic changes in the Hasaki coast since 1987^34^. All survey and research activities are carried out by the Port and Airport Research Institute (PARI). With a tidal range of 1.45 m and an orientation of 59°counterclockwise from the North, this dataset has been instrumental in numerous studies aiming to comprehend the intricate behavior of nearshore morphodynamics^35^. In this study, we utilized a comprehensive 24-year dataset (from 1987 to 2010) of cross-shore beach profile measurements and concurrent offshore wave measurements to identify storm events and subsequently conducted statistical analyses to quantify the influence of key morphometrics on beach recovery.

Hasaki experiences high, mean, and low water levels at 1.25 m, 0.65 m, and −0.20 m, respectively, relative to the HORS datum level (Tokyo Peil +0.687 m)^36^. The median sediment diameter ($d_{50}$) is 0.18 mm^37^. Significant beach erosions are observed in April and during the typhoon season, which typically spans from late August to October, while natural nourishment processes occur during the remaining months of the year^38^.

### Wave data and extraction of storms

**Figure 2:**
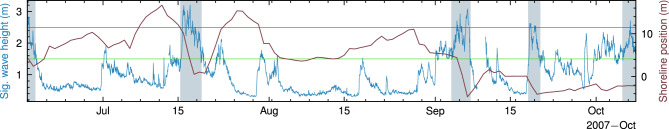
Storm identification based on the two threshold approach. The threshold of 2.5 m was employed to identify storm events and 1.5 m was employed to calculated the storm duration. Shoreline variation with the presence of storms and recovery in post-storm period are also shown here. Consecutive storm occurrence damaging the shoreline recovery is visible in September, 2007.

The mean significant wave height and period over the 24-year period were 1.20 m and 7.87 s, with standard deviations of 0.61 m and 1.91 s, respectively. A graphical illustration showing the variation of annual wave conditions from 1987 to 2010 can be found as Supplementary Fig. S1 online. A site-specific, significant wave height, $H_{S}$, -based storm identification approach was employed as proposed by Thilakarathne et al.^33^. The criteria involved a minimum $H_{S}$ value of 2.5 m and a minimum duration of 6 h, with $H_{S}$ exceeding 1.5 m being considered as the storm duration^13,39^ (Fig. 2). Utilizing the hourly $H_{S}$ data from HORS, a Python module was developed to identify storm events, resulting in 347 storm events. Following the initial identification, the selected storm events underwent additional refinement to exclude consecutive storm occurrence within 10 days. This step was crucial to avoid potential disturbances to the beach recovery process. Additionally, negative erosional storm cases (shoreline moving seaward), where storm events occasionally resulted in accretionary instances, were filtered out. This refinement process was in line with our specific focus on short-term recovery from erosional events. Out of the 347 initially identified storm events, 176 cases showed no consecutive storm occurrence within 10 days. Once the shoreline accretionary events were removed, 125 storm events were available for the analysis.

### Beach profiles and profile categorization

Daily beach profiles were meticulously measured using a cross-sectional profile along the HORS pier, with 500 m wide cross-sections taken at 5 m intervals^36^. The nearshore bathymetry in the longshore direction is almost uniform at the HORS pier; therefore, the impact of longshore sediment transport was not considered in the present study.^40^ In line with the methodology proposed by Thilakarathne et al.^33^, we adopted a shoreline-based profile characterization approach, recognizing the significance of the shoreline position in nearshore profile changes. The HORS profiling, spanning from −115  to 385 m, was adjusted based on the post-storm profile shoreline position, set at 0. This adjustment resulted in a fixed cross-shore length of 420 m, extending −80 m landward and 340 m offshore. The ensuing 420 m-wide cross-sectional profiles were subsequently categorized into three zones: the beach zone, inner zone, and outer zone, taking into account the long-term morphological characteristics at Hasaki (a detailed description is available in Thilakarathne et al.^33^). Furthermore, the profile categorization based on sandbar characteristics as proposed in Fig. 5 of Thilakarathne et al.^33^ was adapted. This categorization includes unbarred profiles, inner sandbar profiles, outer sandbar profiles, and double sandbar profiles. Sandbar identification in the present study was achieved through a Python algorithm designed to detect bar crests and troughs. To ensure the identification of prominent bar formations, minimum height thresholds of 0.25 m and 0.50 m were applied for inner and outer sandbars, respectively, aiming to disregard insignificant bar inclusions in the analysis.

### Beach resilience number

The beach resilience number (BRN) was introduced as a metric to quantify the recovery potential of diverse beach profile patterns. Eichentopf et al.^41^ employed an 18-day post-storm period to detect consecutive storm occurrences at Hasaki. We initially considered 7-day, 10-day, 14-day, and 21-day intervals and compared the occurrence of sequential storms and shoreline recovery measurements for a better statistical approach. We then chose a 10-day threshold, considering the observed recovery patterns at Hasaki. This 10-day selection provided an undisturbed period from consecutive storm occurrences and a significant dataset for our statistical analysis of beach resilience. BRN is defined as the ratio between the shoreline recovery after 10 days and the shoreline erosion during the storm event:

3 $$\begin{aligned} BRN = \frac{BR}{dSL} \end{aligned}$$

Here, BR represents the beach recovery after 10 days (measured from the post-storm shoreline position), and dSL signifies shoreline erosion after the corresponding storm event (measured from the pre-storm shoreline position). For example a BRN value of 1 indicates full recovery of the shoreline to initial state. Additionally, a negative BRN signifies negative recovery, indicating further instances of erosion during the post-storm period.

**Figure 3:**
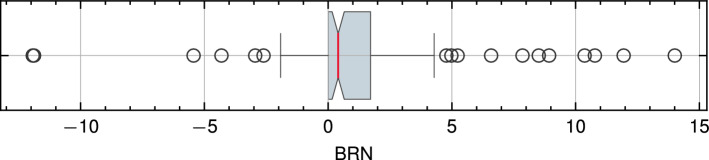
Distribution of BRN values for the 125 storm dataset selected after filtering out consecutive storm occurrences and accretionary cases. The Interquartile Range Technique was employed to detect outliers in the Beach Recovery Number (BRN) distribution for the final storm data filtering, resulting in the selection of the final 104 storm dataset for analysis.

Since the BRN is a measure of beach recovery relative to shoreline erosion, excessively large BRN values might not provide meaningful insights. Consequently, outliers were identified and removed using Interquartile Range Technique^42^ from the BRN dataset as shown in Fig. 3, resulting in a refined dataset of 104 storm cases for the beach resilience analysis.

### Summary of morphometric and hydrodynamic indicators

Table 1 provides a comprehensive summary of all the morphometric and hydrodynamic indicators examined in the feature importance analysis. The careful selection of these indicators aimed to represent profile characteristics spanning from the backshore area to offshore sandbar formations. In addition, due consideration was given to past research on crucial morphometrics. While the sediment size, $d_{50}$, was omitted, the foreshore slope is expected to serve as a representative indicator of sedimentologic characteristics. Fig. 1b shows inner and outer zone definitions based on the foreshore boundary, average breaking point, and closure depth at the study site. Extracted from daily profile data, selected sandbar characteristics are illustrated in Fig. 1c, providing a clear outline of the definitions employed in this study.

**Table 1:** Summary of the morphometric and post-storm 10-day hydrodynamic indicators employed in the present beach study.

Notation	Indicator	Max, min, and mean (± std)	Description
sl	Shoreline position ( m)	24.21, $-29.26$, $-1.73$ (±12.01)	Shoreline of the post-storm beach profile
i$_v$	Inner zone sediment volume $(\textrm{m}^{3}\, \textrm{m}^{-1})$	487.98, 213.83, 398.94 (±55.78)	Volumetric sediment deposition in the inner zone relative to the datum profile, which is based on the lowest cross-section elevation measurements
o$_v$	Outer zone sediment volume $(\textrm{m}^{3}\, \textrm{m}^{-1})$	616.58, 209.03, 445.28 (±73.55)	Volumetric sediment deposition in the outer zone relative to the datum profile, which is based on the lowest cross-section elevation measurements
sp	Foreshore slope	0.1254, 0.0113, 0.0262 (±0.0189)	Tangential slope of the foreshore zone (vertical height/horizontal width)
i$_h$	Inner sandbar height (m)	2.1, 0.25, 0.89 (±0.46)	Sandbar formations in the inner zone (20 m to 170 m) are characterized based on Fig. 1b
i$_l$	Inner sandbar distance (m)	170.0, 70.0, 135.94 (±27.46)	
i$_d$	Inner sandbar water depth (m)	4.03, 1.28, 2.3 (±0.63)	
i$_{ls}$	Inner sandbar landward slope	0.0323, 0.001, 0.0107 (±0.0083)	
i$_{ds}$	Inner sandbar seaward slope	0.0582, 0.0122, 0.0316 (±0.01)	
i$_h$	Outer sandbar height (m)	2.67, 0.61, 1.34 (±0.49)	Sandbar formations in the outer zone (170 m to 340 m) are characterized based on Fig. 1b
o$_l$	Outer sandbar distance (m)	335.0, 175.0, 238.85 (±44.98)	
o$_d$	Outer sandbar water depth (m)	5.19, 2.23, 3.46 (±0.73)	
o$_{ls}$	Outer sandbar landward slope	0.0445, 0.0035, 0.0172 (±0.0089)	
o$_{ds}$	Outer sandbar seaward slope	0.0459, 0.0, 0.0229 (±0.0117)	
h$_m$	Mean $H_s$ (m)	1.93, 0.48, 1.08 (±0.3)	Average of the significant wave heights ($H_s$) during the 10-day recovery period
h$_d$	Standard deviation of $H_s$ (m)	0.77, 0.07, 0.36 (±0.13)	Standard deviation of the significant wave heights ($T_s$) during the 10-day recovery period
t$_m$	Mean $T_s$ (s)	9.88, 4.52, 7.81 (±0.87)	Average of the significant wave heights ($H_s$) during the 10-day recovery period
t$_d$	Standard deviation of $T_s$ (s)	3.04, 0.59, 1.53 (±0.46)	Standard deviation of the significant wave periods ($T_s$) during the 10-day recovery period

Clear definitions of the morphometric indicators are graphically illustrated in Fig.1b, c.

### Feature importance analysis

In the present study, we conducted a feature importance analysis using a combination of XGBoost^30^ models and the SHapley Additive exPlanations (SHAP) algorithm^31^. XGBoost^30^ is a widely used boosting tree-based machine-learning algorithm. SHAP^31^ is a game theory-based feature explanation method, represented by Eq. (4):

4 $$\begin{aligned} \Phi _i = \frac{1}{M!} \sum _{R} \left[ E[f(x) | x_{S_i^R \cup \{i\}}] - E[f(x) | x_{S_i^R}] \right] \end{aligned}$$

where, $\Phi _i$ denotes the Shapley value assigned to feature *i*. The term $E[f(x)|x_{S_i^R \cup {i}}]$ signifies the expected model output for the *R* case when feature *i* is included in the model prediction, while $E[f(x)|x_{S_i^R}]$ signifies the expected model output for the *R* case when feature *i* is excluded from the model prediction. This process of inclusion and exclusion for each indicator forms the foundation of SHAP for quantifying feature importance. Moreover, *M* corresponds to the total count of potential feature combinations, coalitions. This methodology aligns with the approach employed by Thilakarathne et al.^33^, which focused on quantifying feature importance in beach susceptibility.

Initially, we trained four separate XGBoost models using 18 indicators (Table 1) as inputs and the Beach Resilience Number (BRN) as the output. These trained models formed the basis for explaining the influence of each indicator, encompassing 14 morphometric and 4 hydrodynamic variables, on BRN, and consequently, on beach recovery. With the trained models in hand, we employed the SHAP algorithm to explain both local and global interpretations of feature importance. Local interpretations provide insights into how each input variable influences individual predictions, while global interpretations provide a broader perspective on the overall impact of an indicator by averaging the absolute SHAP values across a particular dataset. In the present study, we have four datasets based on the profile pattern. Thus, global interpretation values are calculated as the average of the absolute local interpretations across the profiles within each of the four profile patterns.

As the first phase of feature importance analysis, based on the global interpretation values for each indicator, we selected key indicators with a significant influence on beach recovery, quantified as BRN in this study. This initial selection phase aimed to identify the most impactful indicators, guiding our subsequent analyses. Subsequently, we refined our analysis by training new four XGBoost models for each of the profile patterns using only the selected significant indicators as inputs. These refined models enabled us to investigate deeper into the specific contributions of each selected indicator to the output variable, BRN. By applying the SHAP algorithm once again to these models, we gained further insights into the relative importance of each indicator in predicting the output variable, both locally and globally. This employed feature importance analysis provided a comprehensive understanding of the role played by different indicators in deciding the beach recovery in the 10-day post-storm period.

## Results and discussion

### Feature importance analysis

**Figure 4:**
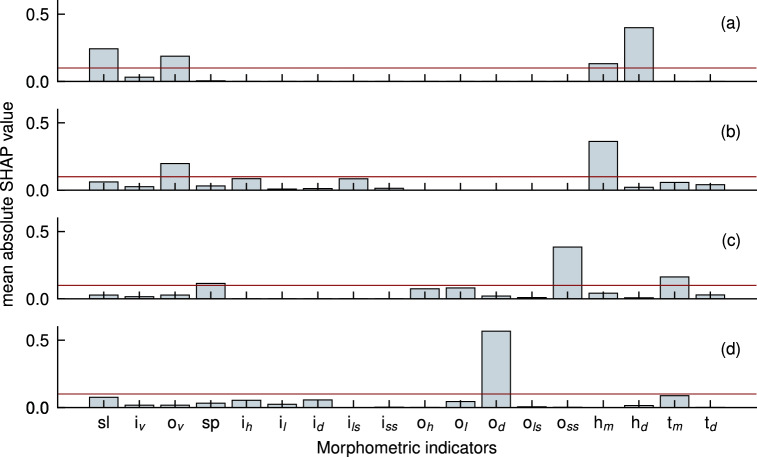
First phase of feature importance analysis using global interpretations of the indicators for the Beach Recovery Number values. Sub figures denote: (**a**) unbarred profiles, (**b**) inner sandbar profiles, (**c**) outer sandbar profiles, and (**d**) double sandbar profiles. A threshold of 0.1 was used to identify the significant indicators as proposed by Thilakarathne et al.^33^.

Normalized SHAP global interpretation values for each indicator across the four profile patterns are shown in Fig. 4. These values were normalized by dividing the sum of their global interpretation, allowing for comparisons within a profile pattern but not across different patterns. The results reveal that key indicators vary depending on the profile pattern, underscoring the influence of the beach profile transformation stage on recovery. Profile patterns signify distinct stages of beach transformation, aligning with the discussions by Sunamura et al.^17^. The inclusion of the last four indicators in our field data captures hydrodynamic conditions in the 10-day post-storm period. In contrast, Phillips et al.^43^ used correlation coefficients to assess the impacts of sandbar location and the rate of cross-shore sandbar migration but considered longer recovery periods of 7, 14, 30, and 60 days, including the presence of consecutive storm occurrences. However, our study, focusing on specific morphometric indicators, was constrained by the presence of consecutive storm occurrences, limiting the post-storm period duration to 10-days. Also, in the initial stage of our study, we tried to use correlation coefficients to explain complex nearshore processes and they were not statistically and theoritically satisfying.

The first phase of the feature importance analysis aimed to identify critical indicators in each profile pattern for use in the subsequent local interpretation phase. Utilizing a 0.1 threshold for each profile pattern yielded distinct indicators for different profiles. Out of the 18 indicators, eight were selected based on their global interpretations concerning BRN. Specifically, for unbarred profiles, the chosen indicators included shoreline position, outer zone sediment volume, mean and standard deviation of wave height in the post-storm period. Inner sandbar profiles featured selected indicators of outer zone sediment volume and standard deviation of wave height in the post-storm period. Outer sandbar profiles were characterized by one morphometric indicator, the seaward slope of the outer sandbar, and the mean value of wave period in the post-storm period. Finally, double sandbar profiles only exhibited one indicator significantly related to beach resilience, which was the outer sandbar depth.

In the second phase of the feature importance analysis, using the selected 8 indicators as inputs, we focused on local interpretations to showcase the variation of importance of each selected indicator in each post-storm recovery case, which was decided based on the post-storm profile pattern. Local interpretations explain the model’s predictions for individual inputs, hence the variation of indicator importance with indicator value can be quantified.

**Figure 5:**
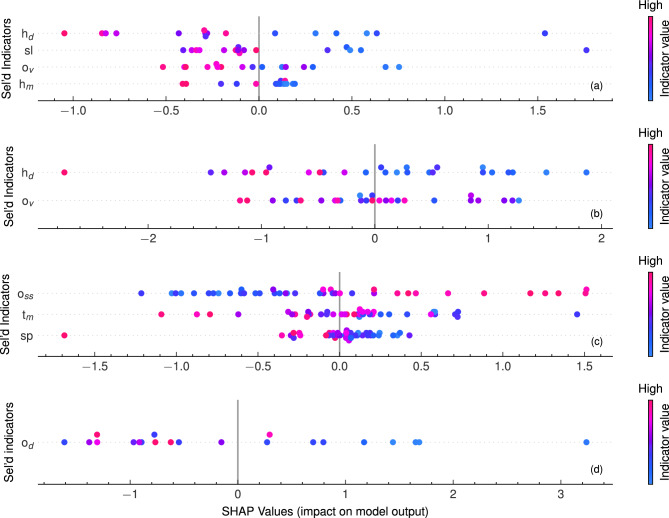
Second phase of feature importance analysis using local interpretations of the indicators for the beach recovery number values. Sub figures denote: (**a**) unbarred profiles, (**b**) inner sandbar profiles, (**c**) outer sandbar profiles, and (**d**) double sandbar profiles. Each color bar denotes the range of indicator values, with blue hues representing smaller values and red hues indicating larger values.

Figure 5 shows the local interpretations for the selected indicators for each profile pattern for the immediate post-storm profiles. A clear variation in the SHAP values, ranging from negative to positive, was observed across all four profile patterns. These SHAP values indicate the contribution of each indicator to the Beach Recovery Number (BRN) for each of the post-storm shoreline change case while the indicator values also changing. These SHAP values indicate the contribution of each indicator to the Beach Recovery Number (BRN) for each post-storm shoreline change case. Each color bar denotes the range of indicator values, with blue hues representing smaller values and red hues indicating larger values. Positive SHAP values suggest significant contributions to positive BRN, indicating resilient beach systems, while negative SHAP values indicate relevant indicators contributing to further erosional cases in the beach zone. For example, in Fig.5a, higher values of $h_d$ represented in red correlate with a negative impact on recovery, due to negative SHAP values, whereas lower values represented in blue contribute positively to beach recovery due to positive SHAP values. In unbarred and inner sandbar profiles, wave conditions had a significant influence on recovery, with severe wave conditions characterized by higher wave heights and periods in the 10 days resulting in negative SHAP values, signifying reduced BRN values and lower resiliency in beach profiles.

### Hydrodynamic condition on beach recovery

Recognizing the significant influence of hydrodynamic variables on nearshore morphology changes, we assessed the impact of wave action during the 10-day post-storm period on beach recovery, as quantified by BRN. The last four indicators among the 18 considered represented the wave condition for each profile pattern. The mean significant wave height ($H_S$) values for unbarred, inner sandbar, outer sandbar, and double sandbar profiles were 1.16 m, 1.12 m, 1.09 m, and 1.00 m, respectively. Similarly, mean wave period ($T_S$) values were 7.90 s, 7.85 s, 7.98 s, and 7.43 s across these four profiles. Among the 104 post-storm cases, 32 exhibited mean $H_S$ values exceeding the long-term average, while 25 displayed negative BRN values, indicating further erosional cases. Interestingly, only 12 of these 25 cases had higher $H_S$ values than the long-term average, suggesting that wave conditions alone do not determine post-storm changes. However, when the mean $H_S$ during the post-storm period exceeded 1.60 m, negative BRNs were more prevalent. Furthermore, it was observed that the majority of positive BRN cases, 78.48 %, occurred under milder wave conditions, reinforcing our approach to exclude consecutive storm occurrences.

A graphical illustration showing the distribution of wave height ($H_S$) and wave period ($T_S$) for the complete dataset, storm conditions, and post-storm periods for each of the profile patterns can be found as Supplementary Fig. S2 online.

### Profile patterns on beach recovery

In Fig. 5a, the local interpretations of each selected indicator for the beach recovery (BRN) of unbarred profiles after 16 storm cases are presented, focusing on how individual post-storm beach morphometrics contribute to beach recovery, either positively or negatively. In the absence of a sandbar, the probability of wave breaking significantly diminishes, allowing larger waves to directly impact the foreshore. This, in turn, hinders post-storm recovery, resulting in less resilient beach profiles. Key morphometrics controlling recovery include shoreline and outer zone sediment volume, where higher values exhibit negative SHAP values, indicating obstructing the recovery. Specifically, the standard deviation of $H_S$, $h_d$, emerges as the most important indicator, shadowing the impact of morphometrics in this context. The accumulation of sediment in the outer zone contributes to recovery during milder post-storm periods. The variation of SHAP values for different shoreline positions (sl) in Fig. a shows that larger shoreline values (seaward shoreline positions) lead to relatively less resilient profiles. Although the magnitudes of SHAP values are not cogent, this suggests that largely accreted profiles are more prone to erosion even under average wave conditions, indicating lower resilience. Unbarred profiles often represent the endpoint of long-term sandbar migration, where most of the sediment is located on the beach, making the beach zone sediment-rich but more susceptible to erosion.

It is needed to highlight that the landward migration process of the sandbar during the spring plays a critical role in ensuring a complete subaerial beach recovery over the mild wave period in the summer, particularly in the context of summer profiles with larger sediment accretions, mostly resulting in unbarred profiles. As highlighted by Ruiz de Alegría-Arzaburu and Vidal-Ruiz ^44^, the recovery period is significantly influenced by seasonal wave climates, a factor not directly addressed in this study.

In Fig. 5b, the local interpretations of each selected indicator for the beach recovery (BRN) of inner sandbar profiles after 27 storm cases are presented. These local interpretations signify the contribution of each selected indicator to BRN, indicating that, similar to the unbarred profiles, wave conditions also exert significant control over recovery for inner sandbar profile patterns. Thus, for this dataset, beach recovery appears to be influenced by the wave condition during the post-storm period. The outer zone sediment volume, $o_v$, emerges as the only morphometric indicating its importance on short-term beach recovery. Although a higher sediment budget in the outer sandbar, $o_v$, appears to impede recovery during the post-storm period, unclear distributions of SHAP values hinder drawing definitive conclusions from this evidence. However, the impact of wave conditions is evident, as higher wave height standard deviations during the post-storm period prevent recovery.

Figure 5c shows the local interpretations of each selected indicator for the beach recovery (BRN) of outer sandbar profiles after 40 storm cases. Outer sandbar profiles were the predominant category among the 104 datasets for post-storm recovery indicators, offering substantial and satisfactory data for conducting the feature importance analysis. Unlike the other three patterns, a clear impact of significant wave height, $H_s$, on the BRN was absent. The variation of the only hydrodynamic indicator, $T_S$, with the SHAP values did not show a strong correlation, and its mean during the post-storm period of outer sandbar profile patterns (7.98 s) was not significantly different from the long-term mean (7.87 s).

When considering morphometric indicators, the seaward slope, $o_{ss}$, and foreshore slope, *sp*, appeared to exert a significant impact on beach recovery, hence, the predominant characteristics of beach resilience. A clear impact was observed between higher $o_{ss}$ and beach recovery, making it the most important morphometric indicator for outer sandbar profiles. This observation is evident in the first row of Fig. 5c, which shows positive SHAP values for higher seaward slopes of outer sandbars. The impact of sandbar slopes on recovery, and beach morphodynamics in general, is an area that has not received significant attention. However, our study considered this aspect and found that the seaward slope of sandbars plays an important role in recovery for outer zone sandbar profile patterns. The local interpretations were clear: steeper sandbars have a positive impact on recovery, while milder slopes have the opposite effect. This means that outer sandbars with steeper slopes are less stable, more prone to erosion, and provide a higher sediment supply, facilitating quicker recovery. The impact of the other chosen morphometric indicator, *sp*, identified in the initial phase of the feature importance analysis, appeared to have a lesser effect on BRN, with SHAP values ranging between $-0.5$ to 0.5. We included it as an indicator, especially as it represents the sedimentological characteristics of the beach and surf zone. Various discussions on the relationship between foreshore slope and sediment size are available^34,37,45,46^. Reis and Gama^47^ have presented a correlation between these two factors based on the constructal law:

5 $$\begin{aligned} \beta = \left( \frac{a}{2}\right) ^{0.67}B^{-1.33}d^{1.33} \end{aligned}$$

where *a* is a wave parameter, *B* is related to the water height, and *d* is the sediment diameter. This relationship indicates that higher slopes represent steeper beaches, a claim also made by Wright et al.^1^.

Figure 5d shows the local interpretations of the only indicator selected for beach recovery, BRN, of double sandbar profiles after 21 storm cases. In the global interpretation shown in Fig.4d, the water depth at the outer sandbar, $o_d$, emerged as significantly important in controlling beach recovery. Shallower water depths at the outer sandbar suggest that waves break even for lower wave heights during the recovery period, disturbing the outer zone sandbar and bringing sediment to the beach under milder wave conditions, facilitating easier recovery. With normalized importance exceeding 0.5, $o_d$ was identified as the sole indicator for double sandbar formations, given that these profiles encompass all 18 indicators. Such a high global interpretation importance, surpassing 50%, provides statistically robust evidence.

Upon closer examination of the local interpretations of $o_d$ for the recovery mechanism, it became apparent that lower water depths were associated with quicker recoveries in the beach system, indicative of more resilient beach profiles. However, the impact of larger water depths on recovery was less clear, with higher values showing negative SHAP values (Fig. 5d). Lower water depths at the outer sandbar profiles facilitate sediment transport, with sand from the bar system moving ashore^48^. The complete restoration of the beach to its pre-storm state depends partly on the successful completion of each preceding stage^3^.

## Conclusion and future work

In the present study, we conducted a detailed statistical analysis of the short-term beach recovery process using a dataset of 104 storm events, with a focus on understanding how recovery varies based on different beach profile patterns. Building upon our previous work^33^, which concentrated on beach susceptibility, we employed several methodologies established in that study to identify storms, profile patterns, and cross-shore zones. Investigating 14 morphometric parameters within four distinct profile patterns (unbar, inner, outer, and double sandbar profiles), in the present study, we aimed to explain and interpret their impact on short-term recovery dynamics. Key influential morphometric features for each of the four profile patterns were identified as shoreline position and outer zone sediment volume significantly for unbarred profiles, outer zone sediment volume for inner sandbar profiles, seaward sandbar slope and beach slope for outer sandbar profiles, and water depth at the outer sandbar for double sandbar profiles. Most importantly, our findings underscored the significant contribution of beach profile patterns to the recovery process, as these patterns represent different stages in the long-term transformation of beach profiles. This study addresses a significant gap in the literature by providing a comprehensive analysis of short-term beach recovery.

Results further highlight the role of wave conditions in controlling beach recovery following extreme storm events. We hypothesize that the insights gained from this study can serve as a foundation for future research endeavors, providing a basis for understanding and analyzing beach resilience variations through the proposed Beach Resilient Number (BRN). Moreover, our observations indicate that water depth at the outer sandbar crest plays a crucial role in governing the recovery process. This aligns with the established understanding that the outer sandbar acts as a sediment source, expediting beach recovery. Additionally, the seaward slope of the outer sandbar emerges as a key factor influencing recovery; steeper slopes appear to yield the beach more fragile, yet contribute to sediment availability for recovery. Therefore, the combination of low water depths and higher seaward slopes contributes to overall beach profile resilience.

While the present study examines the importance of considering beach profile patterns in recovery processes, certain limitations provide insights for future research recommendations. The analysis employed a relatively small dataset size due to the storm data filtering conditions, including requirements for the absence of consecutive storm occurrences and significant erosions during the storms. To enhance the robustness of the proposed BRN and methodology, we recommend the use of larger datasets for validation in future work. Furthermore, we believe that the methods developed in this study are transferrable to various sandy beaches, as evidenced by our satisfactory apprehension of beach recovery dynamics even with a relatively modest dataset. Future research can build upon these insights to advance our understanding of beach recovery and enhance the applicability of the proposed methodologies across diverse coastal environments.

### Supplementary Information

Supplementary Figures.

## Data Availability

Any requests for the dataset of storm and cross-sectional profiles at HORS must be directed to the fourth author, Masayuki Banno.

## Code availability

The codes used to identify storms, BRN calculations, and feature importance analysis are available upon reasonable request from the corresponding author. The codes are also intended to be made accessible at https://github.com/SalikaThilakarathne.
