# Supplementary material for: Identifying key morphometrics to post-storm beach recovery through explainable AI
Source: Sci Rep. 2024 Jun 20;14:14261. doi: 10.1038/s41598-024-64023-6 (PMC11190179; doi:10.1038/s41598-024-64023-6)
Supplement: Supplementary file 1 — Supplementary Figures. [file 41598_2024_64023_MOESM1_ESM.pdf]

Supplementary Information

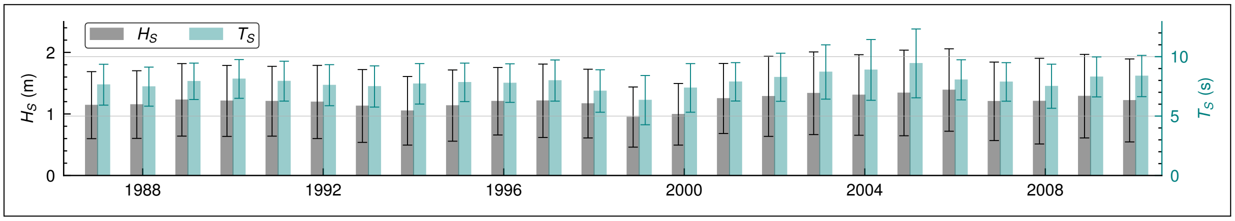

Fig. S1. Variation of annual significant mean wave height (H_S) and wave period (T_S) from 1987 to 2010.

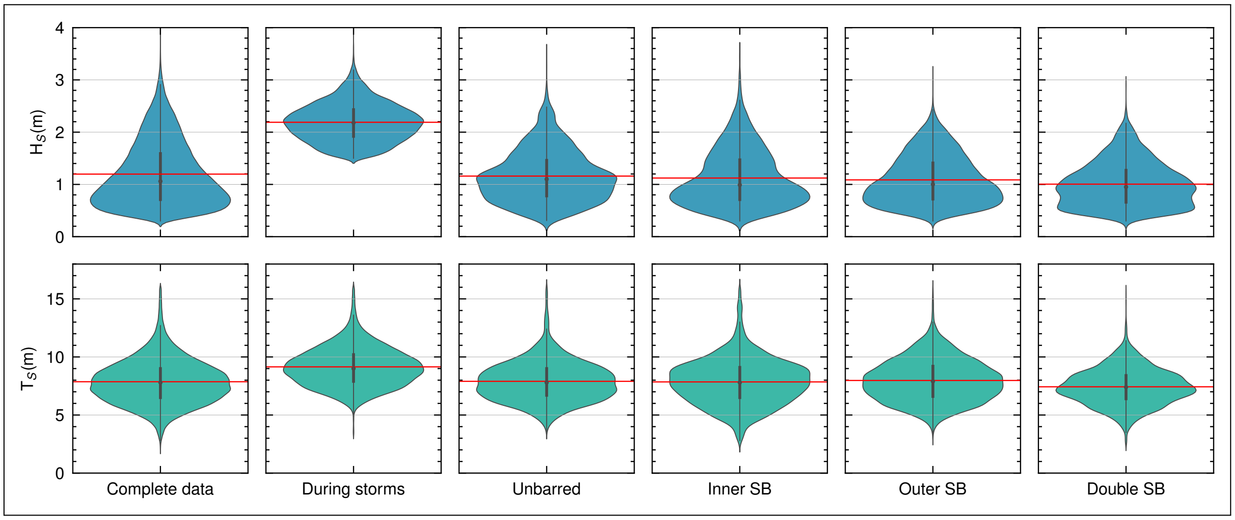

Fig. S2. The distribution of wave height (H_S) and wave period (T_S) for the complete dataset, storm conditions, and post-storm periods for each of the profile patterns.
